# The corticocancellous press fit iliac crest bone dowel for recalcitrant scaphoid nonunion: analysis of union rate and clinical outcome

**DOI:** 10.1007/s00402-023-04846-6

**Published:** 2023-04-03

**Authors:** Ruth Christine Schäfer, Andreas Nusche, Anna Einzmann, Claudius Illg, Adrien Daigeler, Katarzyna Rachunek

**Affiliations:** Department of Hand, Plastic, Reconstructive and Burn Surgery, BG Unfallklinik Tübingen, Eberhard Karls University Tuebingen, Schnarrenbergstr. 95, 72076 Tuebingen, Germany

**Keywords:** Scaphoid, Nonunion, Scaphoid reconstruction, Wrist surgery, Corticocancellous bone graft

## Abstract

**Introduction:**

Scaphoid nonunion after failed primary treatment remains challenging particularly when entailed by bone loss, avascular necrosis or deformity. We describe a scaphoid augmentation and fixation technique for cases of recalcitrant nonunion after screw placement by autologous press fit corticocancellous dowel. This study aims to provide reliable data on clinical and radiological outcomes and to contextualize in the face of other treatment options.

**Material and methods:**

The study included 16 patients with recalcitrant nonunion of the scaphoid. All patients received screw removal and scaphoid reconstruction by a dowel shaped non-vascularized corticocancellous bone graft from the iliac crest facilitating packing of the screw channel. Bone union, the scapholunate**,** radiolunate and intrascaphoidal angles were evaluated on X-ray and CT images, range of motion noted. Additionally grip strength, DASH and Green O’Brien scores were obtained from eight patients.

**Results:**

A union rate of 73% was noted after mean follow-up of 54 months. After revisional reconstruction of the scaphoid an extension–flexion rate of 84% of the healthy side was noted while pronation-supination reached 101%. DASH score averaged at 2.9, rest pain on a numeric rating scale was 0.43 with 99% peak grip force of the healthy side.

**Conclusion:**

In complex cases of revisional scaphoid nonunion after screw placement, the corticocancellous iliac crest pressfit dowel is an option for augmentation and stabilization of the scaphoid by preserving the articular surface.

**Level of evidence:**

IV, retrospective case series.

## Introduction

Scaphoid fractures account for 60% of all carpal fractures and 10% of hand fractures [1]. Depending on fracture location, displacement and delay in treatment, some of these fractures progress to nonunion, occurring in approximately 10% of cases [2]. The natural history of scaphoid nonunion was described by Mack et al. [3] and Vender et al. [4] as wrist arthritis and carpal collapse. For more than 30 years, the recommendation of Mack and Vender for scaphoid reconstruction in case of pseudarthrosis has remained valid. However, there is still no consensus on optimal treatment strategies, with multiple graft and fixation methods available [5].

Rates of consolidation for primary scaphoid reconstruction are satisfying, ranging between 79% without hardware fixation and 94% for vascular grafts with Kirschner wire (K-wire) fixation [6]. For revisional surgery in cases of recalcitrant nonunion after previous treatment attempts, union rates range between 50 and 100% for nonvascularized grafts [7–9] and from 0 to 100% for vascularized grafts [10–13].

In cases of previous headless compression screw placement and nonunion, the scaphoid is left with concavities along the screw channel rendering the chances of repeated screw purchase difficult if not impossible. The introduction of scaphoid plate fixation by Ender in 1977 has since evolved, yielding high consolidation rates in mixed primary and secondary reconstruction series [14–16]. Due to intraarticular placement and necessary plate removal in 21% of cases, we evaluated further options. To facilitate packing of a concaved scaphoid and thus contribute to stability, a pressfit corticocancellous bonepeg from the iliac crest has been utilized in our department since 2009. The ‘Arc de Triomphe’ technique described by Fernandez [17] shows similarities with the dowel but the literature thus far lacks data on clinical outcomes. The aim of this study is to analyze results of this novel surgical treatment in terms of union rate, functional outcomes, complications and patient satisfaction to further improve treatment of recalcitrant scaphoid nonunion.

## Materials and methods

Ethics approval from the institutional review board was obtained for this retrospective follow-up study. Between 2009 and 2017, 16 patients having a history of previous headless compression screw placement with or without previous bone grafting were treated with a pressfit corticocancellous iliac crest bone dowel technique for recalcitrant scaphoid nonunion. All patients were treated surgically at the same European Hand Trauma Center, validated by the Hand Trauma Committee of the Federation of European Societies for surgery of the Hand. Due to a later distal radial fracture one patient was excluded from clinical and radiological outcome measurements.

### Surgical technique

**Fig. 1:**
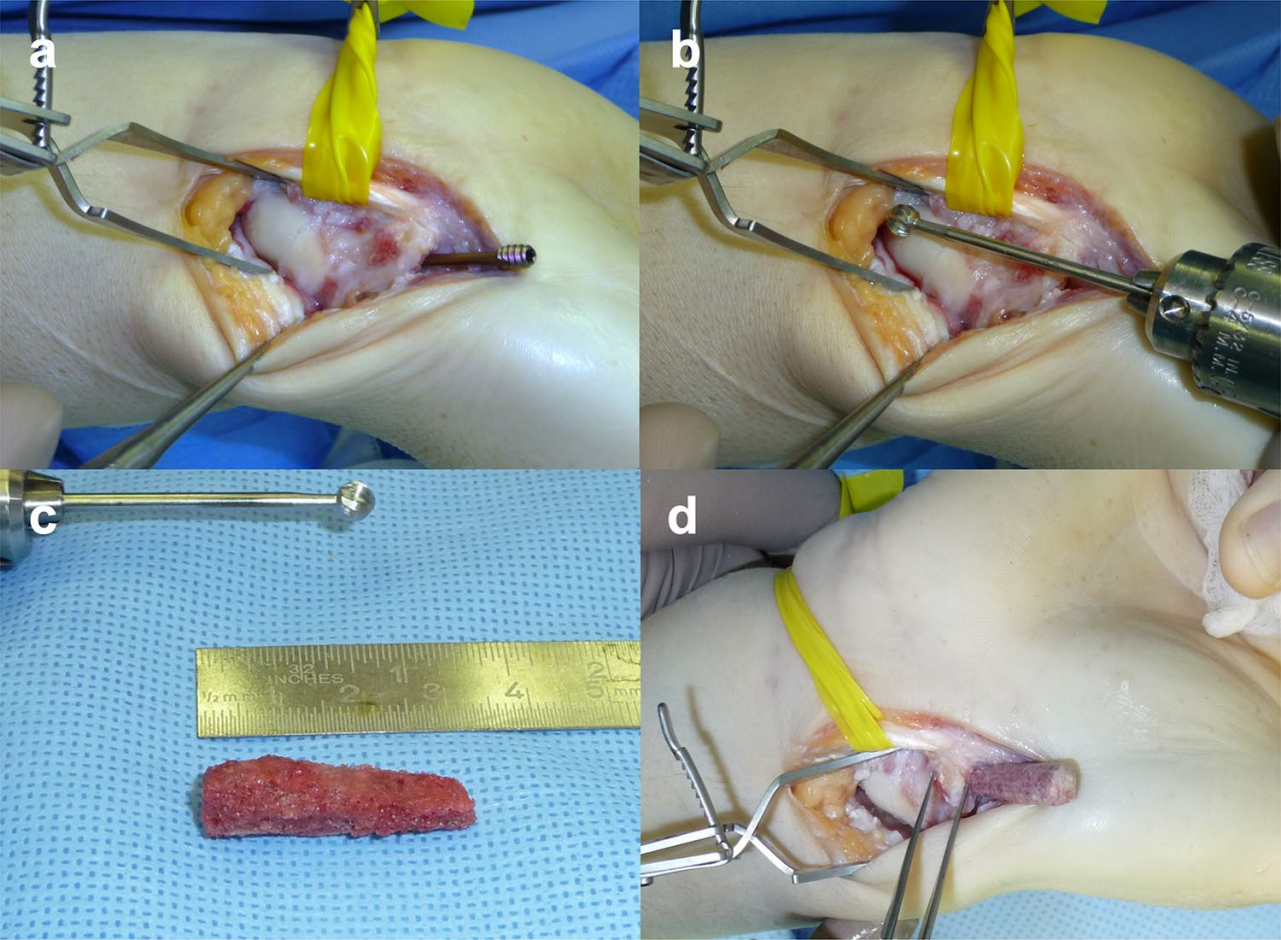
**a** Palmar exposed scaphoid with partially removed headless compression screw from distal pole with proximal nonunion. **b** Debridement of screw channel with drill. **c** Partially trimmed iliac crest dowel to press fit. **d** Iliac crest dowel being driven into the debrided screw channel

Depending on the previous approach for internal screw placement, a palmar or dorsal approach was chosen to expose the scaphoid. After removal of the headless compression screw, fibrous tissue was debrided and the bony defect along the screw channel debrided with a manually driven drill (Fig. 1). The nonunion site and its stability were examined. In cases of tight ‘stable’ pseudarthrosis debridement was achieved through the channel alone to spare the articular surface. In cases of instability, direct debridement of pseudarthrosis was performed until punctual bleeding of both proximal and distal poles. Fragment position was realigned anatomically under fluoroscopy control. To facilitate realignment Linscheids’ maneuver was performed in seven patients while in cases of taut pseudarthrosis lacking misalignment stabilization was pursued directly. Autologous corticocancellous iliac crest bone graft was harvested. The bone graft was then trimmed to a dowel pin shape ranging between 21 and 32 mm in length and a diameter of 3–7 mm with a saw or rose head bur to accurately fit the channel, leaving a strip of cortex along its long axis to maintain stiffness and stability (Fig. 1).

The dowel was driven into the channel by a plunger with great caution taken avoiding a dowel fracture. Further small bone defects resulting from cysts or correction of axis were filled with cancellous bone grafting. In cases of far proximal revisional nonunions or larger defects resulting from fragment reduction, an additional pedicled vascularized bone graft was raised from the radius and grafted [18]. To gain further stability, three patients received an additional temporary K-wire fixation; an insertion of an additional headless compression screw was facilitated in one patient. Postoperatively, wrist and thumb were immobilized with a short arm cast including the metacarpophalangeal joint of the thumb for 10–12 weeks. Radiological union was confirmed by scaphoid visions plain radiographs or computed tomography (CT) scans at approximately 12 weeks after surgery.

### Radiological assessment

Preoperative plain radiographs and CT scans were reviewed for the presence and location of scaphoid nonunion, carpal instability and associated radiocarpal arthritis. The scapholunate angle, radiolunate angle and capitolunate angle were measured on lateral wrist radiographs as described by Larsen et al. [19] to determine dorsal intercalated segment instability (DISI) according to ‘International Wrist Investigator’ Workshop Terminology Committee 2002 [20]. Osteoarthritis (OA) of radiocarpal joints was evaluated using Kellgren–Lawrence Score separately for fossa scaphoidea (FS) and fossa lunata (FL) [21]. Intrascaphoidal angles were analyzed on the coronal and sagittal CT slices [22, 23]. Scaphoid nonunions with normal carpal alignment were classified as Mayo type I and those with carpal instability as Mayo type II [24].

Postoperative analysis of radiographs included union, progression of radiocarpal arthritis and carpal alignment. CT scans were evaluated wherever available [25]. Time to partial union and complete union in weeks was documented. Postoperative scaphoid malunion was diagnosed when a lateral intrascaphoidal angle in CT scans exceeded 45° [26].

### Hand function and scores

Preoperative and postoperative wrist range of motion (ROM) was taken from patients’ clinical records. All patients were invited for clinical follow-up appointment, eight patients failed to respond.

Hand function was measured with a hand rehabilitation system by Biometrics Ltd. (EP11 system) with dedicated E-LINK computer software. Range of motion was measured with a precise electronic goniometer. Standard peak force grip test was measured three times in each (1–5) position with an electronic hand grip dynamometer. The average grip and coefficient of variation percentage were automatically calculated. If the latter exceeded 15%, the grip testing in this position had to be repeated. Force over time was estimated with sustained grip test over 10 s. Time to reach peak force, average over 60%-time, average-to-peak ratio and endurance were further calculated. Standard peak force pinch test was undertaken for key, three-jaw and tip-to-tip positions with three trials per position and consistency of results registered.

The functional outcome was registered by patient self-assessment with the Disability of the Arm, Shoulder and Hand (DASH), SF-36 scores and rest pain together with pain under load on numeric rating scale (NRS; ranging from: 0—no pain, to 10—intolerable pain) within 1 week prior to examination [27, 28]. In addition, the modified Green O’Brien (Mayo) wrist score was used [29].

### Statistical analysis

Statistics were prepared using R programming language and free software environment for statistical computing. Mann–Whitney *U* test was used to compare distribution of the metric variables (age, time between trauma and reconstruction) of patients with and without union after the index operation. To compare the distribution frequency of factors such as smoking, localization of pseudarthrosis (proximal or waist), type of reconstruction and prior operations (osteosynthesis or reconstructions performed) the Fisher’s exact test was applied. To compare ROM pre- and post-operatively the Wilcoxon signed-rank test was applied**.** All tests were calculated two-sided and a statistical difference between the two groups was defined with a significance level of *p* ≤ 0.05.

## Results

Due to exclusion of one patient by recent radial fracture, we retrospectively analyzed 15 patients after revisional scaphoid reconstruction by pressfit corticocancellous iliac bone dowel at a mean clinical and radiological follow-up of 54 (range 6–135) months. All patients were male and average age at the time of operation was 29 years (range 18–41); four patients (27%) were smokers.

Average time between trauma and operation of interest was 17 (range 6–60) months. All patients were right dominant with the left hand affected in eight patients (53%). Detailed demographic data are presented in Table 1.

**Table 1:** Demographic and radiological data

Patient	Age (years)	Profession	Time from injury to surgery (months)	Previous surgery	Additional procedure	Union	Follow-up (months)	Radio-lunate angle		Lateral intra-scaphoid angle	
								pre.	post.	pre.	post.
1	36	Locksmith	9	HCS	K-wire, VRG	No	15	8.7	11.1	42	41.7
2	41	Drywall mechanic	16	HCS	–	Yes	11	19	25	47.4	47
3	25	Industrial mechanic	12	HCS, CBG	VRG	No	6	19	25	44	49.6
4	18	Production scheduler	10	HCS, CCBG	–	Yes	130	13.5	4.1	55	40.2
5	27	Student	36	HCS, CCBG	–	Yes	3	10.7	3.5	50	n.a
6	24	Student	10	HCS	K-wire	Yes	28	n.a	4.9	n.a	35
7	42	Stablehand	6	HCS	–	Yes	10	5.4	5.9	39.3	40.2
8	27	Baker	17	HCS	VRG	Yes	52	17.6	18.9	50.4	50
9	21	Teacher	12	HCS	VRG	Yes	133	10.7	14.9	40.6	28
10	41	Industrial mechanic	24	1.HCS 2. HCS, CBG	–	No	55	17.4	20	42.4	38
11	29	Carpenter	20	HCS, CBG	–	Yes	3	11.8	19.4	39.1	42.9
12	30	Carpenter	60	HCS, CCBG	–	Yes	34	17.1	22.4	38.2	44.7
13	25	Industrial mechanic	28	HCS	HCS, VRG	Yes	27	9.2	13.4	n.a	42
14	19	Automobile sales management	20	HCS, CCBG	VRG	No	135	16	15.3	35.5	37
15	30	Business economist	144	HCS, CCBG	–	Yes	79	11.6	10.8	54	56

*Pre.* preoperative, *post.* postoperative, *HCS* headless compression screw, *n.a.* data not available, *CBG* cancellous bone graft, *VRG* vascularized radial graft, *CCBG* corticocancellous bone graft

Patient-reported outcome scores were retrieved from eight patients. Range of motion after more than 6 months was postoperatively assessed in 14 patients, 8 by clinical evaluation and 6 through chart review.

All patients were reoperated due to failed first procedure. Initially seven patients received headless compression screw fixation in scaphoid fractures, while five patients received pseudarthrosis reconstruction with corticocancellous iliac crest interposition graft along with screw fixation. Previously, two patients had been reconstructed with cancellous bone autograft and headless bone screw and one patient had undergone two scaphoid reconstruction attempts.

In eight cases, the pressfit corticocancellous bone dowel was used as sole bony augmentation and internal fixation for scaphoid reconstruction. Six patients (40%) received a combined operation with a dowel autograft and a vascularized bone graft from the radius: three of them based on 1,2 intercompartmental supraretinacular artery and three from palmar (based on blood supply from Arcus radiocarpalis palmaris or Arcus palmaris metaphysealis). In three patients an additional fixation was performed, in one patient by headless compression screw and in two patients by K-wire.

### Radiological assessment

**Fig. 2:**
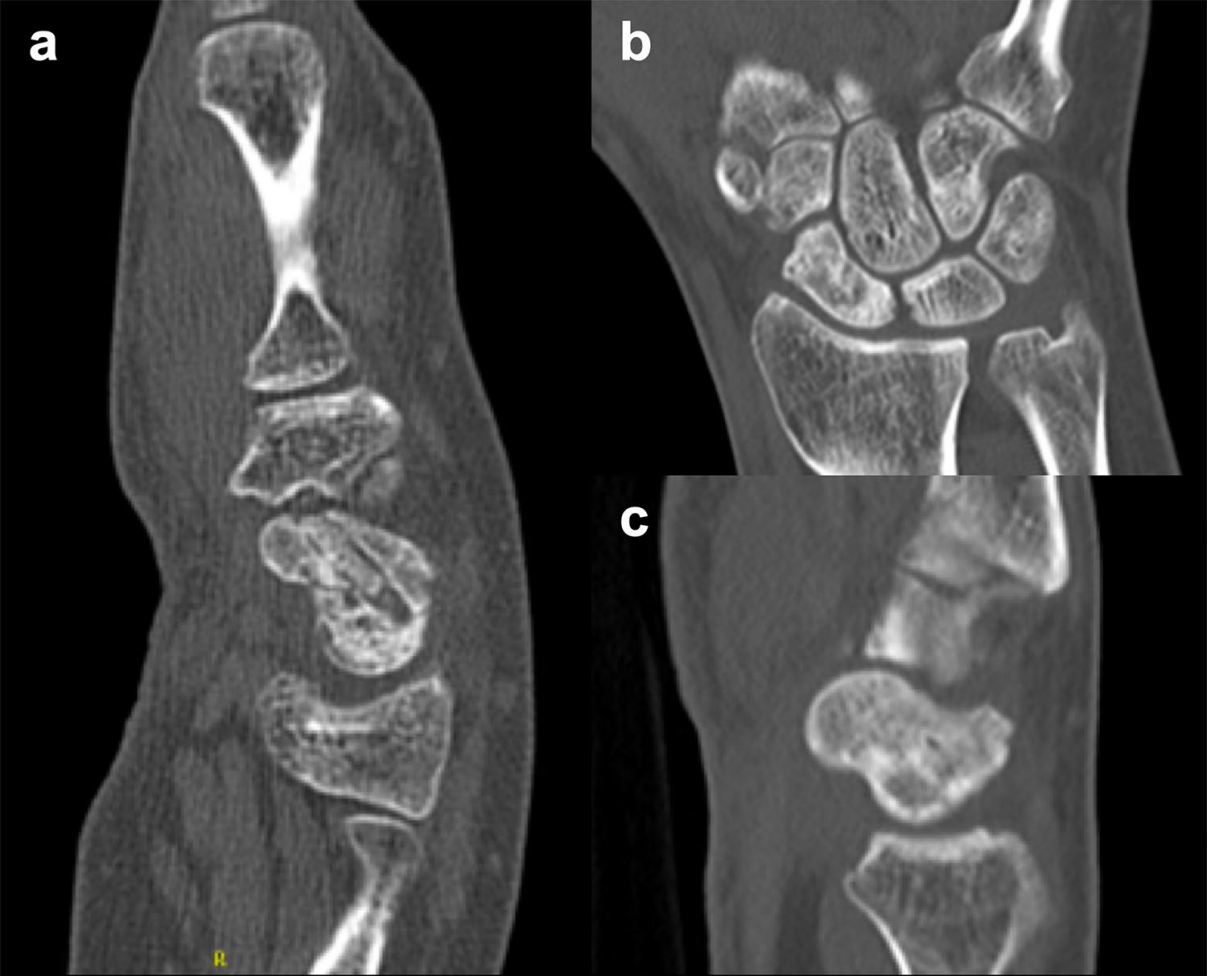
Postoperative CT scans of patient #2. **a** Sagittal view 12 weeks postoperatively with dowel along the scaphoid axis and beginning consolidation. **b** coronal and c. sagittal view 11 months postoperatively with good integration of the dowel to the consolidated scaphoid

**Fig. 3:**
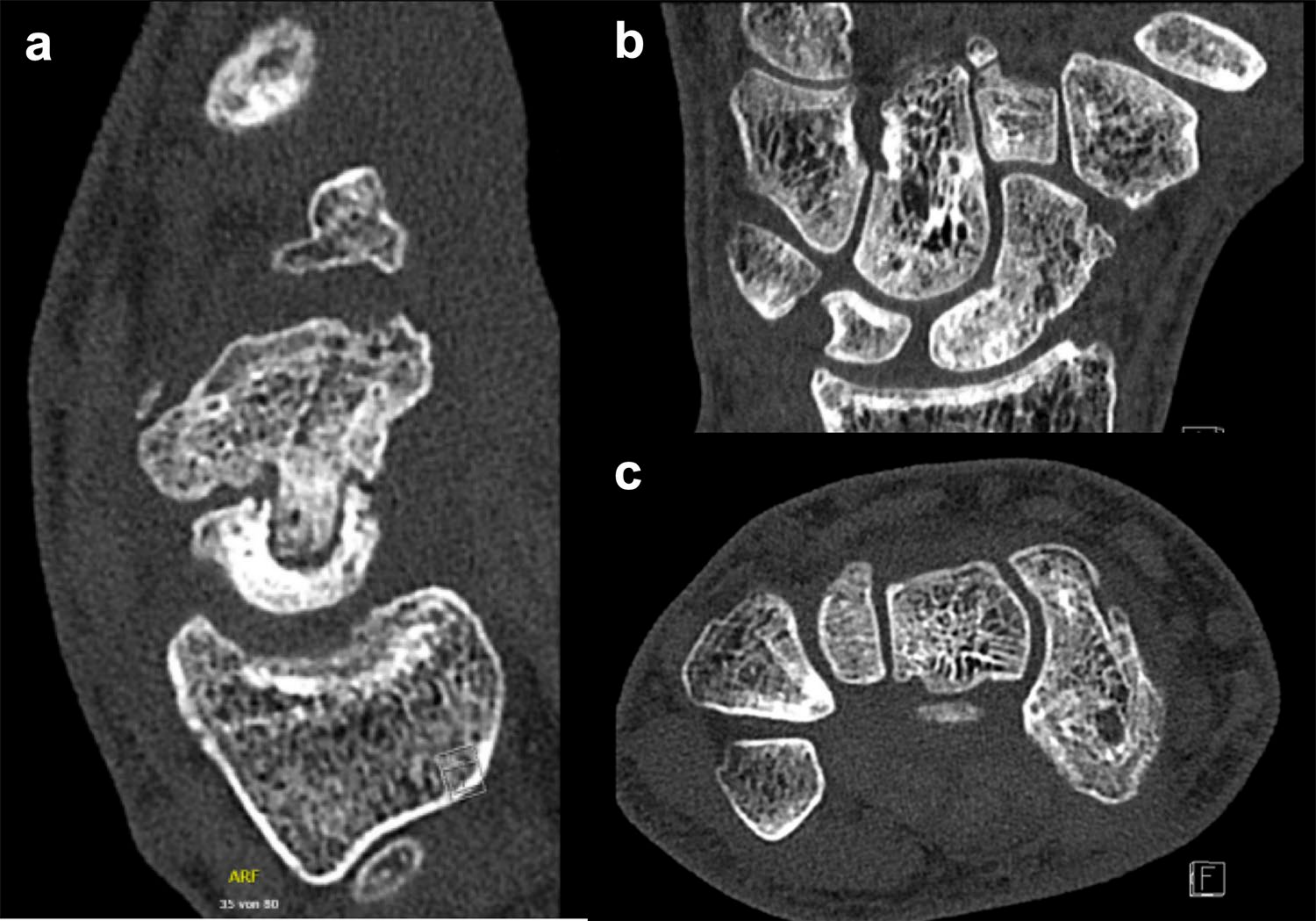
Postoperative CT scans. **a** Sagittal CT scans of patient #14 with failed union after 12 weeks with well notable dowel structure and integration in distal pole. **b** coronal and c. axial scans of patient #8 four years after successful revisional scaphoid reconstruction with dowel

The proximal pole was the site of fracture or nonunion in nine patients (60%) and at the waist in six patients (40%). Scaphoid union by dowel was achieved in 73% (11 patients) of cases. Time to partial union was 7.2 weeks (range 6–12) with time to complete union averaging at 12.9 weeks (range 11–15). Bony consolidation at proximal pole occurred in 56% of all proximal pole revisions and in 100% of nonunions of the middle third; this was, however, not significant (*p* = 0.1). No sclerosis of the proximal pole was seen in preoperative X-rays or CT scans as a sign of avascular necrosis. Examples of dowel impressions in CT scans are given in Figs. 2 and 3.

Age (*p* = 0.95) and time between trauma and index operation (*p* = 0.69) did not seem to influence union rate. The combination of dowel technique with an additional vascularized bone graft from the radius could not enhance the rate of scaphoid union in comparison to dowel technique alone (*p* = 0.14). Three out of six cases of combined dowel and vascularized radial bone grafting failed consolidation. Neither smoking nor the type of previously failed operation yielded any statistical significance.

Preoperatively, Mayo type 2 nonunion was diagnosed in six (40%) patients. Scapholunate angle before operation averaged 58.9° (range 35.7–73.6°) and radiolunate angle 13.4° (range 5.4–17.4). In the postoperative assessment, scapholunate angle averaged 61.3° (range 46–78.8°) and radiolunate angle 14.3° (range 3.5–22.4°). Scaphoid malunion with lateral intrascaphoidal angle > 45° was present in four (36%) patients; three of them had a humpback deformity preoperatively (for detailed intrascaphoidal angles refer to Table 1). Humpback deformity was diagnosed in five patients (33%). All patients with carpal instability postoperatively had previous type Mayo 2 nonunion. Detailed information is shown in Table 1.

According to Kellgren–Lawrence Classification, preoperative OA of first grade in FS was diagnosed in six patients. Progression to third degree OA was evident in one patient after previous failed reconstruction. Overall OA of the FS slightly progressed from an average first-degree OA by 0.07 grades in the postoperative follow-up radiographs. In FL, only one patient demonstrated a doubtful osteoarthrosis first grade which could be excluded intraoperatively. Two radiological patient examples are given in Figs. 2 and 3.

### Scores

Patient-reported outcomes from 8 patients presented a DASH score average at 2.86 (range 0–5.8); DASH score for sport and hobby averaged at 14.3 (range 0–81.3) and Work-DASH at 0.9 (range 0–6.25). The average DASH score in patients with persistent nonunion (*n* = 4) was 3.6 and after a successful treatment 2.3 (*n* = 4). Rest pain of the wrist assessed on NRS averaged at 0.4 (range 0–2), pain under load at 1.7 (range 0–3) postoperatively. Patients with recalcitrant nonunion had higher NRS scores under load at 2.6 compared to 1.25 in consolidated cases; however, these results remained insignificant with *p* = 0.1. In SF-36, physical functioning score averaged at 89.3, role limitations due to physical health 100, role limitations due to emotional problems 100, energy/fatigue 75, emotional well-being 86, social functioning 100 and general health at 91.7. Table 2 presents an overview.

**Table 2:** Patient-reported outcome scores retrieved from eight patients

Parameter				*p* value
Patient-reported outcome	Union	Nonunion	Overall	
DASH score	2.29	2.45	2.36	0.238
Rest pain, NRS 1–10	0.25	0.67	0.43	0.253
Pain under load, NRS 1–10	1.25	2.67	1.67	0.104
SF-36	93.7	83.3	89.3	0.081

For Green O’Brien score two patients yielded an ‘excellent’, three patients a ‘good’ and one patient a ‘fair’ result.

### Functional outcome

**Fig. 4:**
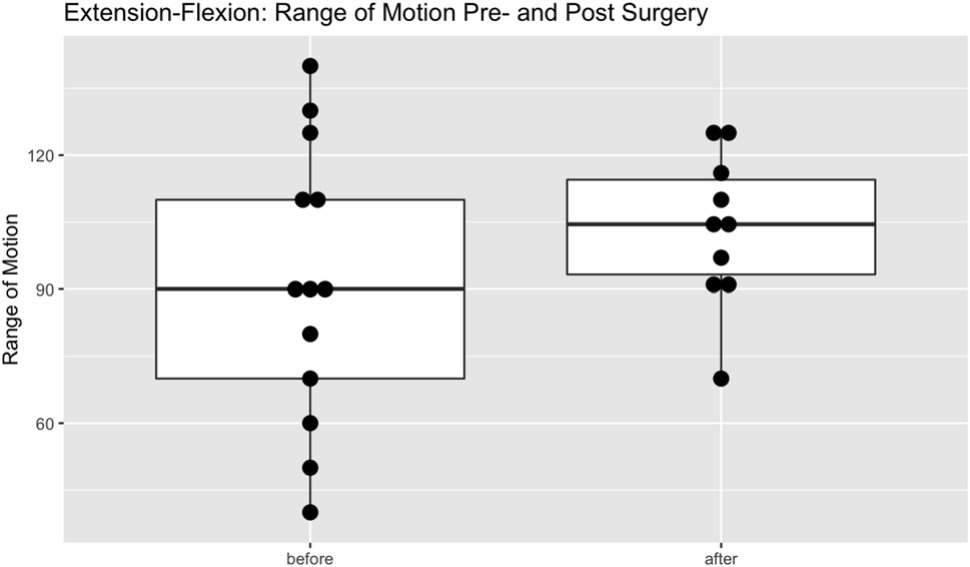
Extension–Flexion ranges of motion in degrees pre- and postoperatively.

The postoperative range of motion in extension–flexion was 106° (range 70°–131°) in the reconstructed wrist reaching 86% of the healthy wrist (average 122°). Radial-ulnarduction reached 84% of the healthy side with an average of 37° (range 30°–55°). Range of motion for pronation-supination improved to 101% of the healthy wrist with 170° average (range 160°–180)°, as shown in Table 3. The postoperative improvement of wrist motion averaged 24° for complete range of motion (cROM), 17° in extension–flexion, with a loss of 2° in radial-ulnarduction and 10° improvement in pronation-supination. However, none of the parameters showed statistical significance. For a detailed view Fig. 4 and Table 3 can be utilized.

**Table 3:** Hand function in range of motions before and after revisional scaphoid dowel reconstruction in fifteen patients

Parameter	Preoperative	Postoperative	*p* value
Complete Range of Motion	290.8°	313.4°	0.097
Flexion in Degrees, mean	45.7°	55°	0.106
Extension in Degrees, mean	45°	50.1°	
Radialduction in Degrees, mean	12.8°	11°	0.857
Ulnarduction in Degrees, mean	26.6°	27.7°	
Pronation in Degrees, mean	80.4°	84.1°	0.075
Supination in Degrees, mean	80.4°	85.6°

Standard peak force grip was only obtained postoperatively and, therefore was compared to healthy side measurements as visible in Table 4. On average, grip force of the affected hand reached 99% (range 72–134%) of the healthy side and 80% (range 49–89%) of the norm, whereas the peak hand grip force of the unaffected hand averaged 89% (range 66–122%) of the norm. The results of a sustained grip force test and of the pinch test are shown in Table 4.

**Table 4:** Hand function testing after revisional scaphoid dowel reconstruction compared to healthy side in eight patients

Parameter	Treated wrist	Untreated wrist	Affected/unaffected ratio (%)	*p* value
Peak force in kg, mean	41.3	41.7	99	0.937
Time to peak in sec, mean	1.1	1	110	0.411
Average over last 60% in kg, mean	31.1	30.5	102	0.841
Key pinch in kg, mean	9.9	10.3	96	0.573
Three jaw pinch in kg, mean	9.7	8.4	115	0.239
Tip to tip position in kg, mean	6.2	5.4	115	0.112

## Discussion

Scaphoid nonunions of the waist and proximal pole continue to challenge the hand surgeon, despite the long history of bone grafting [30–33]. Headless compression screws have improved union compared with historical methods [34]. However, in cases of recalcitrant nonunion after internal screw fixation a channel of resorption along the screw leaves the bone concaved, thus hampering revisional screw placement. Over time the natural history of scaphoid nonunion includes progression to arthritis, as long term outcome studies have shown [35, 36]. To relieve symptoms and improving the range of motion by preventing from progression of osteoarthritis is therefore, the main aim of scaphoid reconstruction [9].

The present study and its limitations need to be considered before interpretation. The retrospective investigation and follow-up account for a lack of valuable pre- and postoperative measurements, such as grip force or patient-reported outcome scores, which limits the ability to draw conclusions regarding improvements from surgery. The limited sample size, operated over the course of 9 years is due to a generally good consolidation rate in primary surgery. Fortunately, only 16 patients presenting such difficult cases of recalcitrant scaphoid nonunion were considered for this technique and, therefore, eligible for the study.

Clinical and radiological follow-up was short in some patients (minimum of 3 months) while the overall follow-up time of 4.5 years has shown consistently stable results even with pre-existing carpal instability Mayo type 2 in 40% of patients.

This study does present promising outcomes for rare and difficult clinical cases and limited treatment options. With a union rate of 73%, our study is in line with previously published data [5–9]. After the first description of scaphoid nonunion treatment by Adams and Leonard [30] it was the Matti Russe technique that opened paths for bone grafting. The headless bone screw introduced by Herbert and Fischer [34] revolutionized treatment by facilitating a minimally invasive percutaneous approach, still considered the standard therapy in fracture treatment. Considering the multidirectional movement of the scaphoid during wrist motion and the importance of preserving articular surfaces, the appeal of this technique becomes easily conceivable. Union rates for revisional scaphoid reconstructions with nonvascularized bone graft vary between 53 and 100%. Bynum et al. (1995) and Carrozzella et al. (1989) reported a union rate of 53% and 60%, respectively, after repeated Russe bone grafting in small patient collectives with few patients receiving additional internal fixation [7, 8]. Studies by Cooney et al. (1984) and Stark et al. (1988) yielded very high success rates with repeat bone grafting using supplemental K-wire fixation [37, 38]. In our study, additional hardware fixation was used in three cases, but no significant improvement in consolidation rate was noted. Nonetheless, we would like to emphasize the importance of rotational stability for scaphoid reconstruction as demonstrated by Jurkowitsch et al. [39]. Neither the compression screw nor the introduced peg can offer complete resistance; thus, we aim to increase screw or K-wire placement to supplement the dowel while sparing the articular surface.

For vascularized bone grafts, a metanalysis by Merrell et al. reported a union rate ranging between 0 and 100% after revisional scaphoid reconstruction [5]. Fernandez and Eggli, reported union in 10 out of 11 patients after a combined operation with an inlay corticocancellous bone graft from the iliac crest and implantation of the second dorsal intermetacarpal artery graft [11]. Significantly, our data could not corroborate an improvement in consolidation after vascularized grafts, but three out of four nonunions occurred in patients with combined iliac crest dowel and vascularized radial graft. Although vascularization of the proximal pole according to clinical aspect was preserved, the vascularized grafts were implemented in cases with palmar defect or far proximal nonunion site. This might explain a higher break down rate. According to a meta-analysis of Pinder et al. [6] including primary reconstructions, K-wire fixation had a higher estimated incidence of union than a screw, when used with vascularized grafts (K-wire, 94% vs. screw, 87%), whereas nonvascularized graft screw fixation had a better union rate than K-wire (K-wire, 88% vs screw, 90%). Union rate without use of hardware reached 79%. Accordingly, Fernandez described an ‘Arc de Triomphe’ graft technique, using a peg inserted into the screw channel together with a second nonvascularized bone bridge fixed with K-wires; he saw screw placement as contraindicated [17].

Improved rotational stability as well as correction of scaphoid malunion by scaphoid buttress plating is evolving. Even in complex nonunions with humpback deformity consolidation rates range from 72 to 100% [15, 16, 40]. Due to impingement, clicking or hardware complications removal is necessary in 21%. In our study, some carpal instabilities and malunions could not properly be corrected using the dowel pin technique. Although Dodds et al. missed describing carpal alignment postoperatively, it seems logical that correction of humpback deformity by buttress plating provides greater stiffness and energy absorption than pure axial stabilization does. For these cases of malunion, scaphoid plating seems favourable.

Although slight signs of osteoarthritis were present in six patients preoperatively, only one patient with successful union showed progression whereas a slight progression was noted in recalcitrant nonunions. Despite the presence of malunion with a lateral intrascaphoidal angle > 45° in four patients and presence of preoperative humpback deformity in three patients, the functional outcomes facilitating return to work were favourable. The improvement of range of motion for wrist extension-flexion and pronation-supination with an average arc of motion in extension-flexion arc of 105° together with an averaged grip force of 99% of the healthy side, makes the revisional scaphoid reconstruction worthwhile. For three patients, a four-corner-fusion was suggested due to imminent risk of progression to carpal collapse. However, none of the patients agreed to the reduction of functionality and up to this point no salvage procedure needed to be carried out [41].

The technique of dowel bone grafting along the screw channel provides bone augmentation as well as axial stability to the scaphoid fragments through insertion by a plunger and press-fitting the channel. Due to bone remodelling and resorption during consolidation this initially rigid plugging might allow some rotational instability. Thus, immobilization by cast or additional fixation seems mandatory for improving consolidation results.

The use of a dowel will not replace the use of headless compression screws where they can still be placed, or the use of plate fixation in cases of malunion or collapse. It rather serves as an additional option in the treatment of recalcitrant nonunions with substantial resorption where further drilling and screw or wire placement might risk fragmentation or the loss of articular surface. Comparable with screw placement, a maximal preservation of articular surface of the scaphoid can be achieved using this technique to maintain the multidirectional movement of the scaphoid. This particular technique offers a salvage option for the scaphoid from dorsal or palmar approaches depending on previous operation and bone deformity.

## Data Availability

Raw data were generated at BG Unfallklinik Tübingen. Derived data supporting the findings of this study are available from the corresponding author R.C. S. on request.
